# Committors without Descriptors

**DOI:** 10.1021/acs.jctc.5c01848

**Published:** 2026-02-11

**Authors:** Peilin Kang, Jintu Zhang, Enrico Trizio, TingJun Hou, Michele Parrinello

**Affiliations:** † Atomistic Simulations, 12478 Italian Institute of Technology, 16156 Genova, Italy; ‡ Innovation Institute for Artificial Intelligence in Medicine of Zhejiang University, College of Pharmaceutical Sciences, 12377 Zhejiang University, Hangzhou, Zhejiang 310058, China; § State Key Lab of CAD&CG, 121451 Zhejiang University, Hangzhou, Zhejiang 310058, China

## Abstract

The study of rare events is one of the major challenges in atomistic simulations, and several enhanced sampling methods toward its solution have been proposed. Recently, it has been suggested that the use of the committor, which provides a precise formal description of rare events, could be of use in this context. We have recently followed up on this suggestion and proposed a committor-based method that promotes frequent transitions between the metastable states of the system and allows extensive sampling of the process transition state ensemble. One of the strengths of our approach is being self-consistent and semiautomatic, exploiting a variational criterion to iteratively optimize a neural-network-based parametrization of the committor, which uses a set of physical descriptors as input. Here, we further automate this procedure by combining our previous method with the expressive power of graph neural networks, which can directly process atomic coordinates rather than descriptors. Besides applications on benchmark systems, we highlight the advantages of a graph-based approach in describing the role of solvent molecules in systems, such as ion pair dissociation or ligand binding.

## Graphical abstract

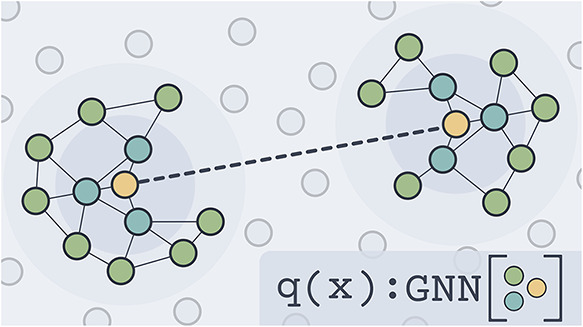

## I Introduction

Atomistic simulations are an indispensable tool in the study of complex physicochemical processes. However, such simulations find one of their limits in the gap between the affordable simulation time and the typically much longer time scale over which many important phenomena like chemical reactions, protein folding, and crystallization take place. Such processes are indeed characterized by rare transitions between metastable states, which are separated by large free energy barriers that act as kinetic bottlenecks hindering sampling. This has been called the rare event problem, and, since the introduction of umbrella sampling some 50 years ago, a multitude of enhanced sampling approaches have been suggested to solve it.^1^

Recently, we have introduced a new enhanced sampling method based on the committor function that greatly alleviates the rare event problem^2^ by promoting extensive sampling of both transition and metastable states, and have started using this approach to solve a number of real-life problems.^2–5^ We recall here that the committor *q*(***x***) is a function of the atomic coordinates ***x*** which, given two metastable states *A* and *B*, gives the probability that a trajectory started in ***x*** reaches *B* without having first passed by *A*.^6^ The committor is arguably the most precise way of describing rare events, since it is a quantity that remains well-defined even if the transition from *A* to *B* follows different competing pathways or passes through an intermediate metastable state. The committor is also believed to be the optimal one-dimensional reaction coordinate.^7^

Unfortunately, if one follows the committor formal definition, a rather expensive trial-and-error strategy is needed for its determination.^8^ Recently, alternative ways have been proposed to learn the committor function from simulation data using machine learning tools in combination with different learning criteria and enhanced sampling schemes.^2,3,9–17^ Such methods have been recently reviewed in ref 18. Our contribution to this area^2,3^ is based on the Kolmogorov variational principle, which is obeyed by the committor^19^ which has been solved in a self-consistent procedure that eventually leads not only to the calculation of *q*(***x***) but to an extensive and balanced evaluation of the free energy equation.

However, like in all variational calculations, the quality of the results depends on the expressivity of the trial function used.^3^ In our initial approach, we have represented *q*(***x***) as a feed-forward neural network *q*(***x***) = *q*_θ_(***d*** (***x***)) whose input is a set of descriptors ***d*** (***x***) chosen to be invariant with respect to the symmetries of the system and whose weights θ are optimized to minimize the functional associated with Kolmogorov variational problem. Despite having proven to be effective in several challenging systems,^2–5^ this approach still relies, at least partially, on the user’s insight for the choice of appropriate descriptors, which, in complex cases, might not be easy to select.

The purpose of this paper is to remove as much as possible this potential obstacle and make the calculation of *q*(***x***) as automatic as possible. To achieve this goal, we parametrize the committor with a geometric Graph Neural Network (GNN),^20^ which can directly use as input the atomic Cartesian coordinates ***x*** while respecting the invariance laws of the system. This architecture has already proven to be useful in building machine-learning potentials^21–23^ and in designing collective variables (CVs).^24–28^ When using a GNN, an atomic system is naturally represented by a graph whose nodes are the atoms and whose connecting edges describe their relationship. Optionally, nodes and/or edges can be assigned attributes that encode information on the system, greatly facilitating the analysis of the results,^27^ especially if an attention mechanism is added to the GNN structure.^29^ Furthermore, GNN architectures can be made invariant or equivariant with respect to the symmetry operations of the system. However, due to their more complex structure, GNN models are computationally more expensive when compared to standard models, with the overall cost increasing with the complexity and size of the graph. To alleviate this limitation, we have also introduced new ways of reducing the computational cost of using a GNN when dealing with reactions in condensed phases.

In the field of enhanced sampling, it is customary to use as a test the conformational equilibrium of alanine dipeptide. We will stick to this tradition and start with the study of this molecule. Having passed the dipeptide test, we will demonstrate the usefulness of this new approach in a number of more physically relevant examples in which the solvent plays an important role. The examples studied are the dissociation of NaCl and of CaCO_3_ in water, and the binding of an organic molecule to calixarene, a simplified but still representative model of drugprotein interaction. All these systems have been studied with other means and thus they provide a good testing ground also for the versatility of the committor since their reactive processes can exhibit multiple reaction pathways and/or metastable intermediate states.^30–34^

## II Methods

### II. A Background

To learn the committor *q*(***x***), we use the Kolmogorov variational principle,^6,19^ which implies minimizing the functional

1 $$K\left[q\left(x\right)\right]={\left\langle {\left|\nabla_{u}q\left(x\right)\right|}^{2}\right\rangle }_{U\left(x\right)}$$

under the boundary conditions *q*(***x***_*A*_) = 0 and *q*(***x***_*B*_) = 1, where ***x***_*A*_ and ***x***_*B*_ denote an initial and final configurations taken from states *A* and *B*, while ***u*** indicates mass scaled coordinates. The average ⟨·⟩_*U*(***x***)_ in eq 1 is taken over the Boltzmann ensemble of the studied atomistic system, which we assume to interact via the potential *U*(***x***).

However, the evaluation of K[q(x)] is in practice far from trivial. In fact, the committor has a step-like structure that raises rapidly from *q*(***x***) ≈ 0 when ***x*** ∈ *A* to *q*(***x***) ≈ 1 when ***x*** ∈ *B*, as it goes through the transition state (TS) region. This makes the term |∇_***u***_ *q*(***x***)|^2^ sharply peaked on the TS region, which unfortunately is hard to sample, since it is seldom visited when dealing with rare events due to the presence of large energetic barriers.

In order to get around this sampling issue,^2,3^ we thus resort to enhanced sampling. In ref. 3, to enhance sampling of the otherwise elusive TS region, we introduced the bias potential $V_{K}\left(x\right)$

2 $$V_{K}\left(x\right)=-\frac{1}{\beta }\log|\nabla q\left(x\right){|}^{2}$$

where β is the inverse temperature. Exploiting the above-discussed localization of the committor gradients, such a bias is able to stabilize the TS region and turn it into a minimum that can be sampled as extensively as a standard metastable state. In addition, to promote also transitions between the states and favor ergodic sampling, in ref. 2, we complemented this approach with a metadynamics-like bias using the on-the-fly probability enhanced sampling^35,36^ (OPES) based on a committor-derived collective variable (CV). Even if the committor is believed to be formally the best possible reaction coordinate, its direct use as a CV presents numerical problems that make such an approach ineffective. For this reason, we make what basically amounts to the change of variable *q*(***x***) = σ­(*z*(***x***)), where σ­(*z*) = 1/(1 + *e* ^–*pz*^) and use *z*(***x***) as CV. Since σ­(*z*(***x***)) a monotonous function, *z*(***x***) encodes the same information as *q*(***x***), but avoids the numerical issues that are a consequence of the sharp behavior of *q*(***x***) and of the fact that, being *q*(***x***) a probability, it can assume values that are smaller than the limit of machine precision.

The solution to the Kolmogorov variational problem is found in an iterative process that starts from an initial guess for the committor. In ref. 3, the initial guess was constructed using data collected in two unbiased simulations performed in the *A* and *B* basins. Such an initial guess does not have any information on the TS states, and as such, this initial guess is not optimal. A better starting guess, which will be used here, is to start the self-consistent procedure, using also data coming from the TS. Such data can be obtained, for example, from metadynamics simulations, even if driven by suboptimal CVs.

One important feature of the committor is that it provides a powerful analysis tool for understanding the reactive processes. One crucial element in this regard is the identification of those configurations defining the transition state ensemble (TSE). Following the spirit of our approach, we have proposed to identify the TSE using the Kolmogorov distribution $p_{K}\left(x\right)$ , defined as

3 $$p_{K}\left(x\right)=\frac{e^{-\beta U_{K}\left(x\right)}}{Z_{K}}\;\mathrm{with}{\;U}_{K}\left(x\right)=U\left(x\right)+V_{K}\left(x\right)$$

This distribution indeed measures the contributions that trajectories passing by a configuration ***x*** bring to the transition rate ν_*R*_, since

4 $$\nu_{R}\propto \int \mathrm{dx}p_{K}\left(x\right)$$

The advantages of using this definition of TSE are discussed in ref. 2 and in Section 3 with examples.

### II. B Machine Learning the Committor with GNNs

As anticipated in the introduction, we change the model used to parametrize the committor and, rather than using a feed-forward neural network with a set of descriptors as previously done,^2,3^ we use a graph neural network (GNN) that directly processes the atomic Cartesian coordinates. In addition, we add to the original loss a few regularization terms aimed at stabilizing and simplifying the training. Apart from these modifications, the procedure is the same as described in our previous work.^2,3^

#### II.B.I Loss Function

The loss function employed here is composed of three terms

5 $$L=\log L_{v}+\alpha_{1}L_{b}+\alpha_{2}L_{r}$$

where the hyper parameters α_1_ and α_2_ regulate the relative strength of the different terms. In the first term, $L_{v}$ is the estimate based on a training set of *N*_v_ configurations ***x***_*i*_ of the functional K[q(x)]

6 $$L_{v}=\frac{1}{N_{v}}\sum_{i=1}^{N_{v}}w_{i}|\nabla_{u}q\left(x_{i}\right){|}^{2}$$

Since most of the time the data will come from biased simulations, a statistical weight *w*_*i*_ is associated with each ***x***_*i*_ so as to give each data its statistically correct contribution. As in ref. 4, we do not insert in the loss function directly $L_{v}$ but its logarithm to improve training stability since $L_{v}$ can vary by many orders of magnitude. This change does not alter the minimum of the functional, since K[q(x)] is positive definite and the logarithm is a monotonously growing function, but avoids numerical problems since K[q(x)] can vary by very many orders of magnitude.

The second term, *L*_*b*_, enforces the correct boundary conditions *q*(***x***_*A*_) = 0 and *q*(***x***_*B*_) = 1

7 $$L_{b}=\frac{1}{N_{A}}\sum_{x\in A}{\left(q\left(x\right)-0\right)}^{2}+\frac{1}{N_{B}}\sum_{x\in B}{\left(q\left(x\right)-1\right)}^{2}$$

evaluated on a labeled data set of *N*_*A*_ and *N*_*B*_ configurations belonging to *A* and *B* respectively. We note that there is no need to introduce additional collective variables to distinguish the metastable states when generating the initial labeled data set, since short unbiased trajectories initiated from either A or B will remain confined to the initial basins in a rare event scenario.

Finally, dealing with the greater expressivity of GNN-based models, we realized that it was better to control the range of accessible *z* values, which in principle could have been (−∞, +∞), and avoid overfitting the metastable states. We thus introduced the regularization term $L_{r}$ to control the range of the *z* value accessible and to make optimization more balanced.

8 $$L_{r}=\frac{1}{N}\sum_{i=1}^{N}{\left[\max\left(0,\left|z_{i}\right|-z_{r}\right)\right]}^{2}$$

where *z*_*r*_ is a user set threshold value and *z*_*i*_ the configurations’ predicted *z* value.

#### II.B.II GNN Model

As GNN architecture, we adopt that of SchNet^37^ proposed by Schütt et al., which provides a good balance between computational cost and expressivity. In addition, to better understand the relationship between neighboring nodes, in examples of the CaCO_3_ dissociation and the ligand binding examples, we have applied a node-level attention mechanism to filter out the less relevant messages coming from neighboring nodes.

SchNet is a message-passing graph neural network designed for atomistic systems, in which the nodes of the graph represent the atoms in the system. To each node is also associated a set of features that are initialized based on the corresponding atom type. Such features are then updated through the network via interaction layers modeled as continuous convolution filters.

In each interaction block, the message passed to the atom *i* from the atom *j* is given by

9 $$m_{\mathrm{ij}}=W\left(h_{j}\right)f_{\theta }^{F}\left(\mathrm{RBF}\left(d_{\mathrm{ij}}\right)\right)\mathrm{with}{\;d}_{\mathrm{ij}}=\left|x_{i}-x_{j}\right|$$

where ***h***_*j*_ is the feature array of atom *j*, ***W*** is a trainable linear transformation, and *f*_θ_^*F*^(·) is a filter network based on the pairwise interatomic distance expanded by a set of radial basis functions (RBF). The sum of messages from all neighbors is then used to update the representation of atom *i*

10 $$h_{i}^{l+1}=h_{i}^{l}+f_{\theta }^{M}\left(\frac{1}{N_{n}}\sum_{j\in N\left(i\right)}m_{\mathrm{ij}}\right)$$

where the *j* index runs over the *N*_*n*_ nodes in the neighborhood N(i) , and *f*_θ_^*M*^(·) is a network that processes the total message received by each node.

Instead, if the attention mechanism is employed, the update function is modified as

11 $$h_{i}^{l+1}=h_{i}^{l}+f_{\theta }^{M}\left(\sum_{j\in N\left(i\right)}\alpha_{\mathrm{ij}}m_{\mathrm{ij}}\right)$$

where α_*ij*_ is the trainable attention score^29^ computed as

12 $$\alpha_{\mathrm{ij}}=\frac{\exp\left(g_{\theta }\left(m_{\mathrm{ij}}\right)\right)}{\sum_{k\in N\left(i\right)}\exp\left(g_{\theta }\left(m_{\mathrm{ik}}\right)\right)}$$

in which *g*_θ_ is a gate network that returns a scalar value for each message incoming to node *i*. To summarize, the incorporation of such an attention mechanism enables the network to assign varying importance to neighboring nodes based on their features and spatial relationships, rather than treating all neighbor messages equally.

As anticipated in the previous section, following ref. 2, we express the committor as *q*(***x***) = σ­(*z*(***x***)), with σ being the activation function described above. More in detail, the value of *z* is computed by first applying a pooling operation (i.e., average) to the node features ***h***_*i*_ of the last GNN layer and then feeding this to a readout network *f*_θ_^*R*^ according to

13 $$z=f_{\theta }^{R}\left(\frac{1}{N_{p}}\sum_{i}^{N_{p}}h_{i}\right)$$

where *N*_*p*_ is the number of nodes involved in the pooling operation. This choice proved to be more stable than what was done in our previous work,^27^ in which the readout function was applied before the pooling operation. In the most general case, the pooling operation runs on all the nodes in the graph, whereas, in the case of the truncated graph discussed in the next section, the pooling operation is applied to a subset of the most relevant atoms only.

#### II.B.III. Improving Efficiency

An important contribution to the overall efficiency and eventual applicability of our approach comes from the way the model input graph is constructed. In fact, one of the main limitations of GNN models is their greater computational cost, which scales rapidly with the number of atoms included in the graph. Often, it is not strictly necessary to include in the graph all the atoms in the system. For example, when dealing with chemical reactions in a solvent, heterogeneous catalysis, or protein ligand binding, only the molecules close to the reactants take an active part in the reaction and should thus be taken into account, whereas the rest of the solvent molecules can be safely ignored.

To take advantage of these considerations, we use the dual neighbor list schematically depicted in Figure 1 for the case of a molecule dissociation in water, which is structured as follows. First, the atoms in the system are divided by the user into two nonoverlapping categories. The reacting atoms, which are always included in the graph and the environment atoms, which are included in the graph only if close to the reacting atoms. In practice, a first neighbor list NL_out_ is built based on a cutoff *R*_*t*_ with respect to the reacting atoms (panel A), then the graph is constructed by associating nodes to all the atoms included in NL_out_ and connecting them with edges according to a second neighbor list NL_graph_ based on a cutoff *R*_*c*_ (panel B). Importantly, the cutoffs are chosen *R*_*t*_ = *R*_*c*_ + Δ_*b*_ to always have a buffer region (Δ_*b*_ > 0) of environment atoms not directly interacting with the system, thus stabilizing the calculation between the neighbor lists updates. This is similar to what is done in molecular dynamics when updating neighbor lists.

**Figure 1:**
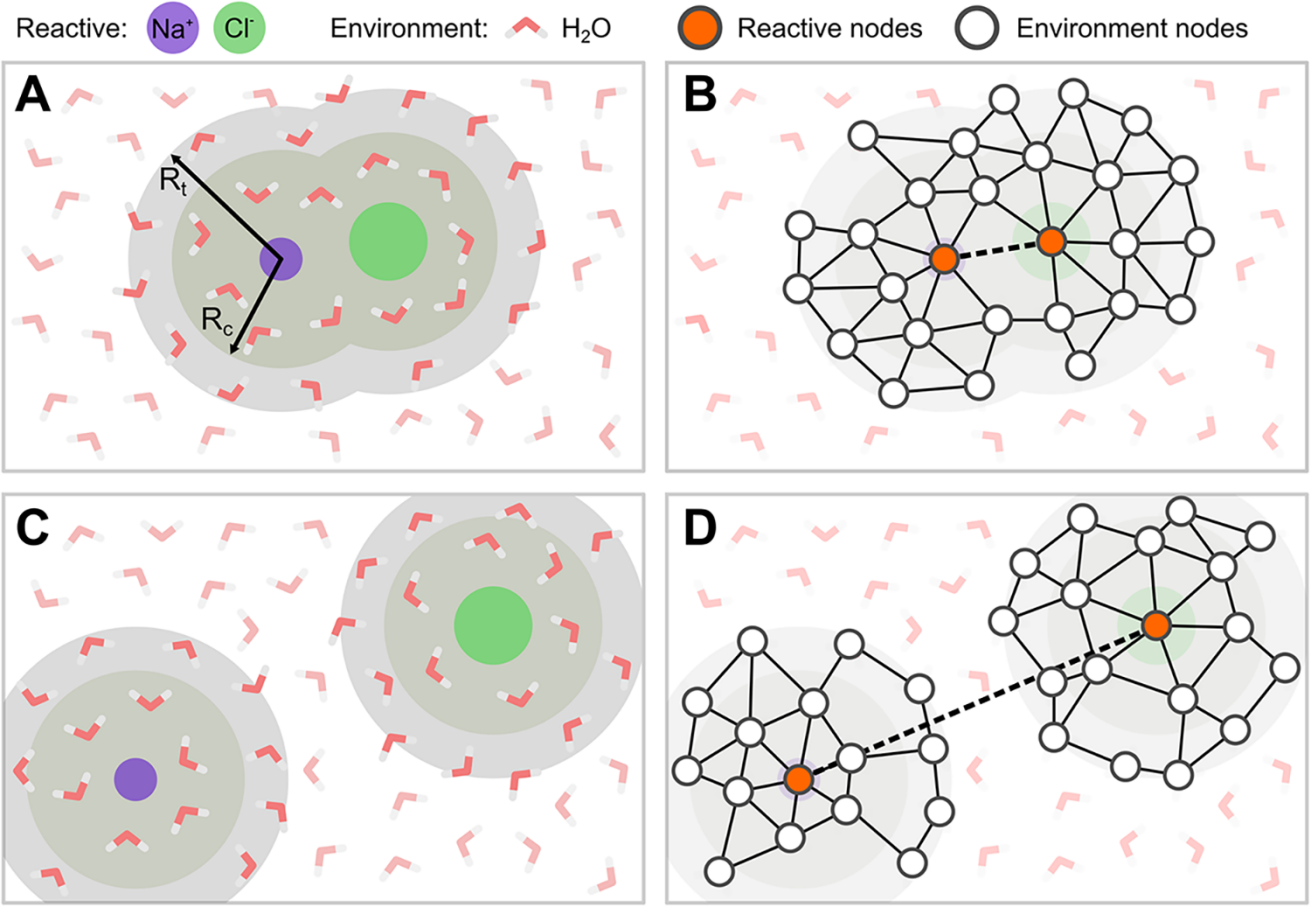
Truncated graph. Schematic representation, using the dissociation of NaCl as an example, of the truncated graph construction to reduce the computational cost of using GNN-based methods when a few *reacting atoms* (NaCl) interact with a large number of *environment atoms* (H_2_O). Only the atoms belonging to the neighborhood defined by the cutoff radius *R*_*t*_ from reacting atoms are associated with graph nodes, whereas the other are neglected (A and C). Such nodes are then connected with edges based on the cutoff *R*_*c*_ < *R*_*t*_ (B and C). To avoid having reacting atoms in disconnected graphs, edges between reacting atoms can be enforced regardless of the distance (D). The whole truncated graph is processed through the GNN model, but only the reacting atoms are considered in the readout function to obtain the final CV output.

To avoid atoms belonging to disconnected graphs based solely on *R*_*c*_, as it could easily happen when two ions or a ligand and a guest are far apart in a solvent (panel C), connectivity between reacting atoms can be guaranteed by defining their interaction based on fixed edges between them (panel D).

The use of this truncated graph approach, already for systems of relatively small sizes, such as the NaCl studied in this paper (i.e., 216 water molecules), we find great computational savings both in the training and the simulation stage.

### II.C GNN Interpretability

The use of a geometric GNN to model atomistic systems brings several advantages in terms of the physical insight that can be extracted once the model is optimized. For example, as atoms are directly mapped to graph nodes, performing a node-level sensitivity analysis can clearly identify which atoms are most relevant for the process in a simple way. Here, we perform such an analysis in two ways. In one, we followed the node sensitivity analysis proposed in ref. 27, in which the modulus of the derivatives of the model output with respect to the Cartesian coordinates of a node is taken as a measure of its relevance. In practice, considering a data set of *N*_*g*_ graphs, the sensitivity *s*_*i*_ of the *i*th node is computed as

14 $$s_{i}=\frac{1}{N_{g}}\sum_{j=1}^{N_{g}}\left|\frac{\partial z\left(G^{j}\right)}{\partial x_{i}^{j}}\right|$$

Where, $G^{j}$ is the *j*th entry of the data set, and ***x***_*i*_^*j*^ is the position of the *i*th node in $G^{j}$ .

Alternatively, when an attention layer is employed, one can use the attention scores α_*ij*_ as a measure of node relevance of the *i*, *j* interaction (see eq 12).

Another important advantage of GNN models is that, starting from simple atomic coordinates, they inherently learn a representation of the system that encodes structural information in the hidden node features of the model. In fact, a distance metric, for instance, the Euclidean distance, can be defined in the latent space identified by such features that can be used to perform unsupervised analysis, for example, based on clustering algorithms or other dimensionality reduction algorithms.

## III Results

The technical details of the simulations, GNN hyperparameters, training procedures and computational cost for the examples presented are provided in the Supporting Information (SI).

### III. A Alanine Dipeptide

In most studies devoted to rare event sampling, the transition of alanine dipeptide in vacuum between the C7_eq_ (*A*) and *C*7_*ax*_ (*B*) conformers is commonly used to demonstrate the effectiveness of new methods. This system is one of the most extensively studied models for rare events and is often characterized by two dihedral angles, ϕ and ψ. Following ref. 3, we begin with unbiased simulation data sampled in the two metastable basins. To build the input for our GNN model, we consider the 10 heavy atoms of the molecule, which serve as the nodes in the input graph.

Using the optimized GNN model, which reaches convergence within 4 iterations (see SI for details), to drive our enhanced sampling simulations, we obtain an estimate of the free energy surface (FES) in close agreement with reference results obtained by biasing the conventional ϕ, ψ. In addition, we can automatically identify the key atoms involved in the transition from the GNN architecture. In particular, a node sensitivity analysis reveals that atoms 3, 4, 5, and 6 contribute most significantly to the learned committor (see Figure 2B). As expected, these are the atoms closely related to the dihedral angles ϕ and θ, in full agreement with previous studies.^3,38^ The two-dimensional committor map projected on two backbone dihedral angles ϕ and ψ and other detailed discussion can be found in Figure S5.

**Figure 2:**
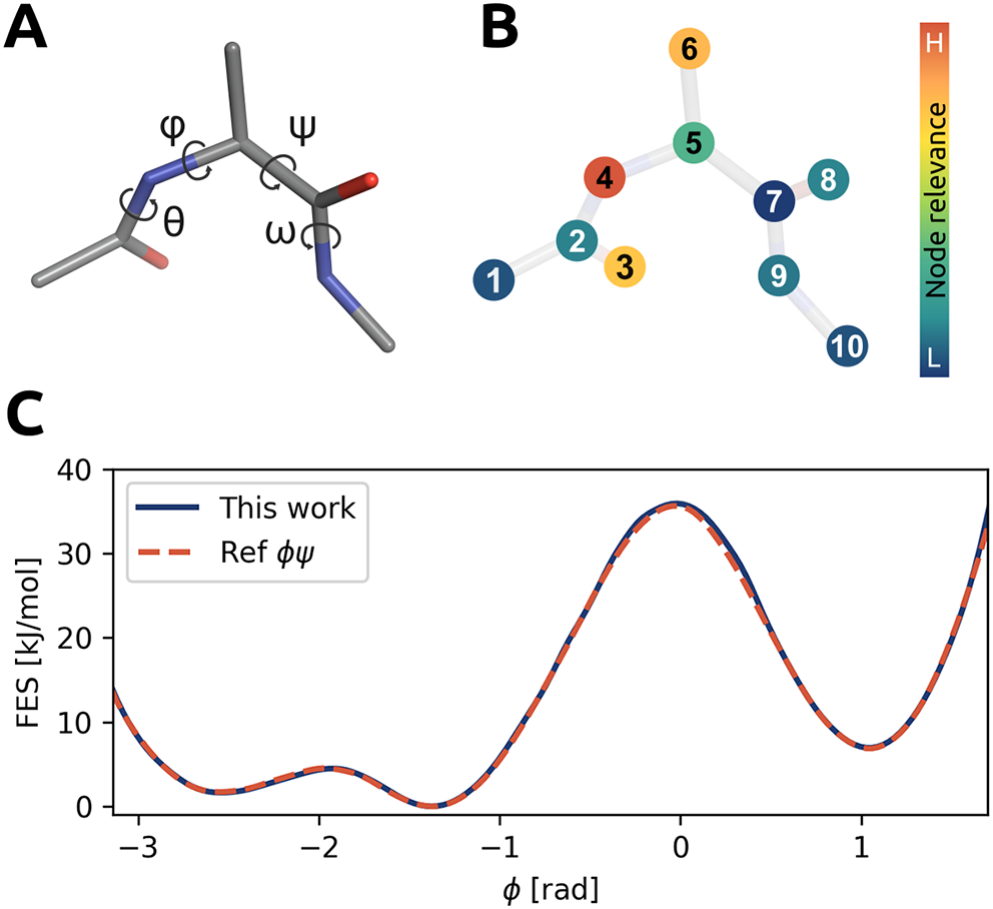
Alanine dipeptide. (A) Relevant torsional angles of alanine dipeptide. (B) Relative relevance, given by the color scale, to the GNN-based committor model of the graph nodes associated with the heavy atoms. (C) Free energy surface (FES) projected along the ϕ torsional angle obtained with the GNN-based setup proposed in this work (blue solid curve) and a reference OPES simulation using ϕ and ψ as CVs (red dashed curve).

### III. B Calixarene

Having paid homage to tradition, we now study the interaction of the G_2_ ligand­(4-cyanobenzoic acid) with an octa-acid calixarene host (OAMe) from the SAMPL5 challenge,^39^ which provides a good example of some features of protein–ligand systems.

Previous studies have shown that the system primarily follows two transition pathways: a “wet” path, in which the process proceeds through the semibound state *B*_w_ before reaching the fully bound state *B*_d_, and a “dry” path, where guest molecules enter and exit the pocket directly. In addition, the wet path has been shown to be dominant even if it involves more intricate dynamics. Here, we shall not repeat the calculation of ref,^2^ but focus only on one of the rate-limiting step, i.e., the transition from the *B*_w_ to *B*_d_ with the purpose of illuminating the role of water in this transition. The results obtained with the setup presented in this work for the whole process are available in the SI, including the overall free energy surface and the estimate for the binding energy.

For the *B*_w_ to *B*_d_ rate-limiting step, our algorithm finds two pathways, one in which the ligand is solvated while at the same time the calixarene is emptied of water and eventually binds to the now dry calixarene (see SI Figure S10). However, as the contribution of this path to the rate is negligibly small (<5%, based on analysis of $p_{K}$ contributions), we consider here only the dominant path (see Figure 3). In this dissociation mode, the water that is trapped in the calixarene in the *B*_w_ state accompanies the ligand to the entrance of the calixarene until it is released into the bulk solvent, leaving room for the ligand to bind to its lowest free energy state.

**Figure 3:**
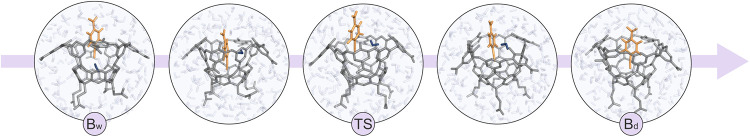
Calixarene. Snapshots of representative configurations along the dominant reaction pathway of *B*_w_ to *B*_d_ in the binding process of the G_2_ ligand (orange) to the OAMe host molecule (gray) in water, grouped according to the *z* value. The water molecules are represented as blue sticks, depicted in transparency when outside the binding cavity and in solid color when inside.

### III. C NaCl

We now discuss the case of NaCl dissociation in water, in which the solvent clearly plays a central role. The contact ion pair (CIP) is our state *A*, while *B* is the dissociated state. To focus on the dissociation process itself, we set an artificial repulsive wall that limits the interionic distance at 6 Å. This implies that in the calculation of the free energy of the *B* state, part of the entropic contribution is missing.

In our previous work^2,3^ and also in the case of alanine dipeptide, we started the committor learning self-consistent procedure with two unbiased simulations started in *A* and *B*, i.e., without any data whatsoever coming from the transition state region. Despite being a procedure of general applicability, this is often an overkill since one can often obtain some information on the TS by running first a metadynamics-like simulation, even if driven by a suboptimal CV. To showcase this strategy, we start with data coming from an OPES simulation in which the interionic distance was used as CV. Even for a simple example like this, this approach allowed reducing the number of iterations needed to reach convergence (see SI, Section S4).

In Figure 4A, we project the free energy surface onto two physically transparent quantities, the ion–ion distance *d*_NaCl_ and the number of bridging water molecules that coordinate both ions at the same time *n*_*B*_.

**Figure 4:**
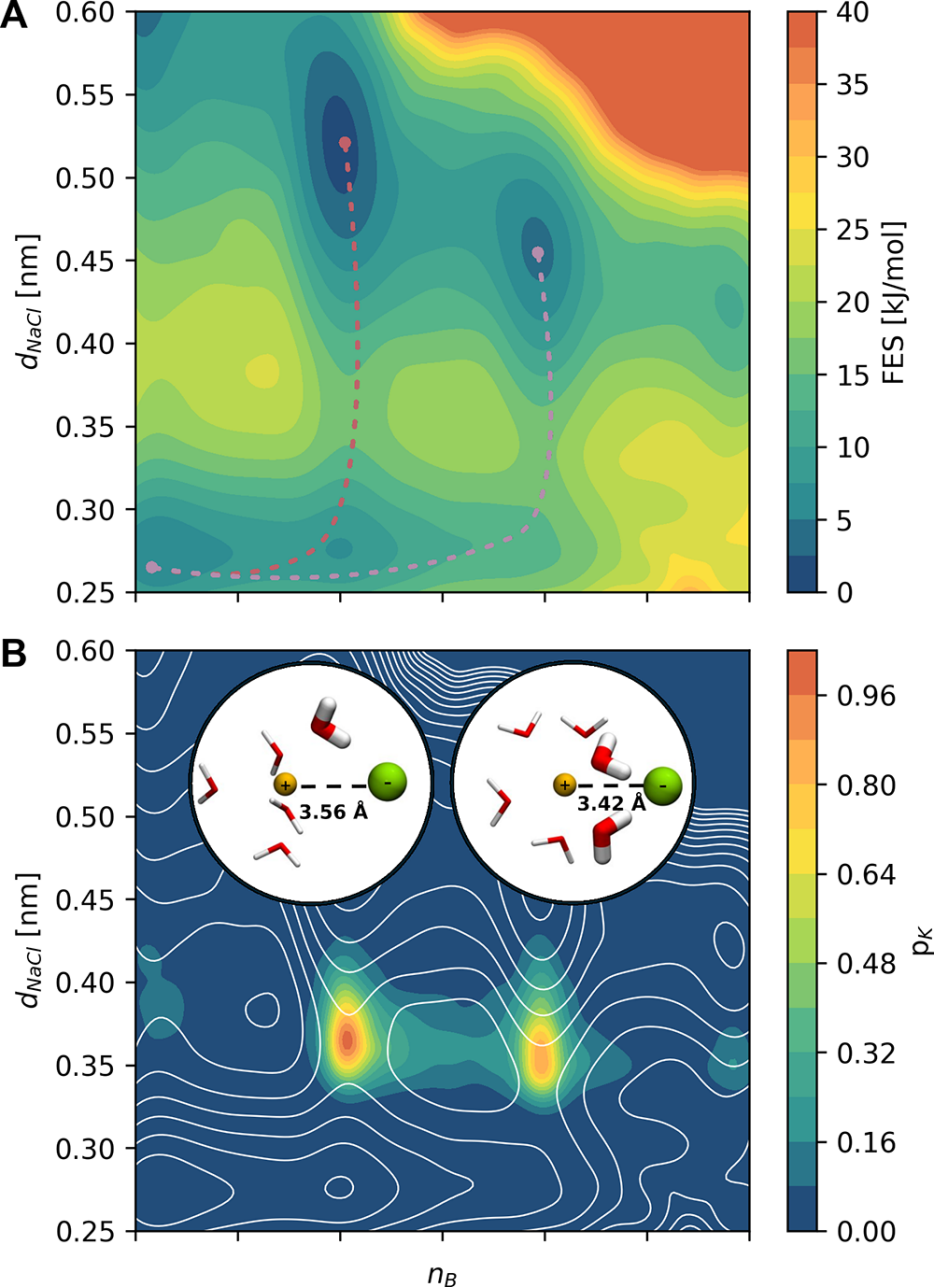
NaCl dissociation. (A) Computed free energy surface (FES), indicated by the color map, in the space defined by the number of water molecules bridging the ions *n*_b_ and by the interionic distance *d*_NaCl_. The two reactive pathways from the associated states are indicated by dashed lines. (B) Distribution of the Kolmogorov probability $p_{K}$ , indicated by the color map, in the *n*_b_ and *d*_NaCl_ plane. The isolines of the free energy of A are superimposed in white as a reference. The insets show representative configurations of the transition states that characterize the two reaction pathways.

Usually, the transition state ensemble is defined as the set of ***x*** for which *q*(***x***) ≈ 0.5, however, as discussed in refs 2,3,5 , it is better to base our analysis on the transition state ensemble as defined by $p_{K}\left(x\right)$ , which gives the probability that a state ***x*** is actually visited along the reactive trajectory. In this case, the $p_{K}\left(x\right)$ distribution has a multimodal structure in which each mode can be classified by the number of water molecules *n*_b_ that are shared by the two ions (see Figure 4B). Using eq 3 and measuring the integrals over the two main peaks of $p_{K}\left(x\right)$ , we can estimate that about 50% of the reactive trajectories pass via the *n*_b_ ≈ 1 transition state and 40% via *n*_b_ ≈ 2. The remaining 10% contribution comes from the minority paths through *n*_b_ ≈ 0 and 3.

For this relatively simple system, the above analysis, based on our physical understanding of the system, we could interpret the results rather straightforwardly. However, in more complex systems, this may not have been so straightforward. For this reason, we show that using the information encoded in the GNN, one could have arrived at the same results in an unsupervised way. Thus, we repeated the $p_{K}\left(x\right)$ analysis, performing a *k*-medoid clustering^40^ using as metric the Euclidean distance between the node features of the last GNN layer (see Sec. II C). We find again that two modes dominate the $p_{K}\left(x\right)$ distribution, which correspond to the two possible screening configurations described above, where the ions are screened by one or two water molecules.

### III. D CaCO_3_

The final test of our method is the study of the dissociation of CaCO_3_ in water, which is another nontrivial example of a chemical process in solution that further showcases the power of the GNN-based approach. For this system, we take as state *A* the contact ion pair in which Ca^2+^ and CO_3_^2–^ form a nearly planar structure close to the gas-phase equilibrium geometry, with the Ca^2+^ ion symmetrically positioned in front of two carbonate oxygen atoms. In such a state, the screening cloud of Ca^2+^ is composed by five water molecules and two carboxylic oxygens. The model potential used at the simulated system size predicts that two major solvation structures (B_6_ and B_7_) are possible. Such structures are almost degenerate: in B_7_ the cation is surrounded by 7 water molecules arranged in a pentagonal bipiramid, while an octahedron of 6 waters forms the solvation shell of B_6_. The fact that the final state is not unique might at first seem like a major problem for a committor based approach, in which the final state has to be specified beforehand, but in fact using just B_7_ as final state we were still able to discover B_6_ and to obtain a free energy surface (see Figure 5A) in agreement with previous metadynamics-based investigations.^31^

**Figure 5:**
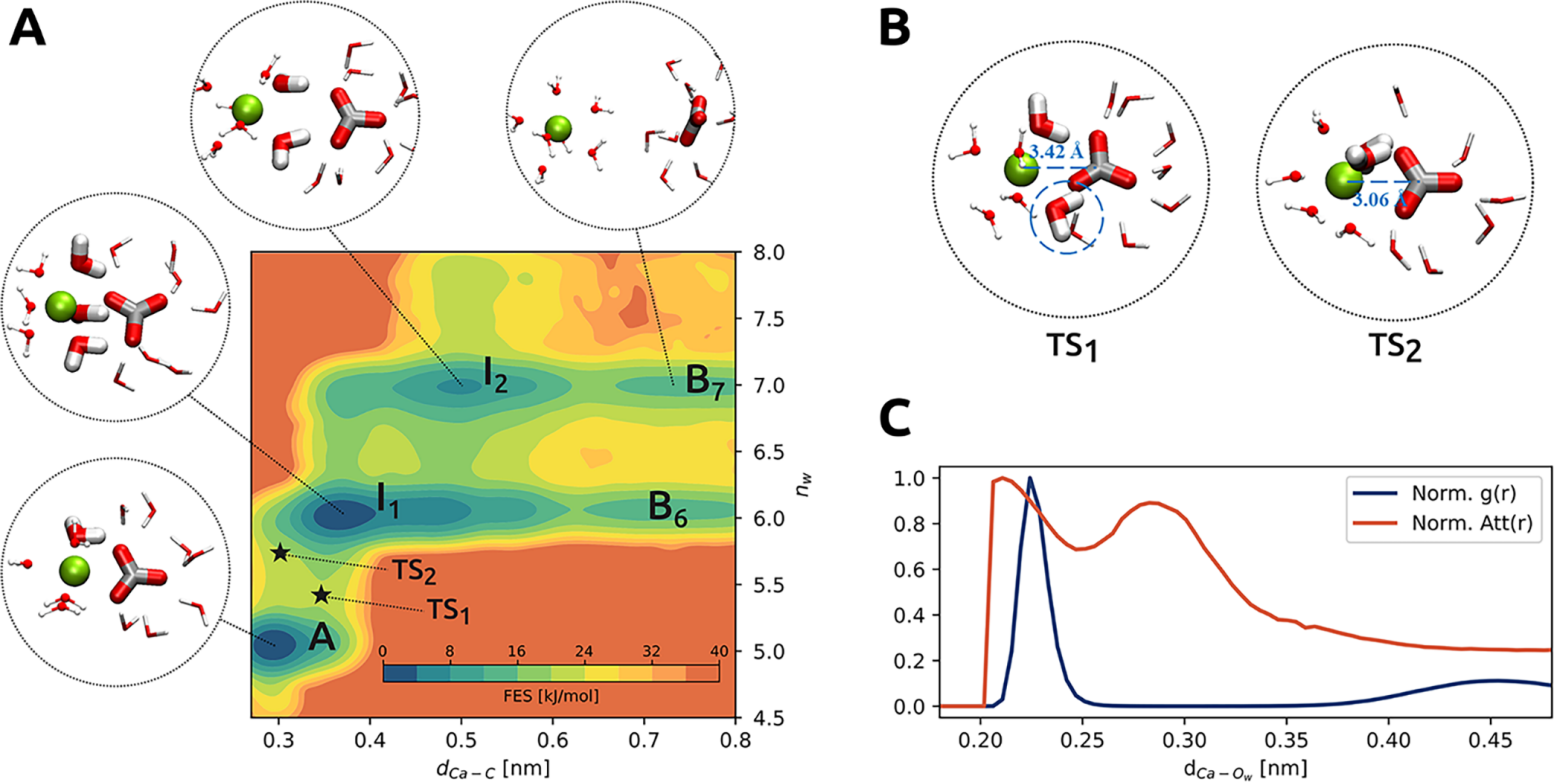
CaCO_3_ dissociation. (A) Free energy surface (FES), indicated by the color map, in the plane defined by the interionic distance *d*_Ca–C_ and the number of solvating water molecules around the Ca^2+^ ion *n*_w_. The metastable states are indicated on the FES, and representative snapshots are provided in the insets. The position of the transition states is indicated by a star. (B) Medoid configurations for the two transition state ensemble clusters TS_1_ and TS_2_. (C) Average attention scores Att­(*r*) of messages from water oxygen nodes to the Ca^2+^ node as a function of the interatomic distance *d*_Ca–O_w__ (red line). The radial distribution function of water oxygens with respect to Ca^2+^ is reported as a reference (blue line). The two curves are both normalized to be in the range (0,1).

The resulting FES (see Figure 5A) shows that, starting from state *A*, the system passes via an intermediate state *I*_1_ in which the number of solvating water *n*_w_ = 6. From this state, two reactive paths branch out leading to the two different final states described above. The intermediate state *I*_1_ is characterized by the loss of the planarity characteristic of *A* and the replacement of one of the coordinating carbonate oxygen atoms by one water molecule. From *I*_1_, one can go either directly to B_6_ or to B_7_ via the intermediate *I*_2_ (*n*_w_ = 7), which corresponds to a near-dissociated configuration in which Ca^2+^ is fully coordinated by seven water molecules and the now distant carbonate anion no longer directly contributes to its screening. However, in this state, the solvation shells of the two ions still share two water molecules.

The rate-limiting step of the whole process is the transition from *A* to *I*_1_, which can take place in two ways, as revealed by the bimodality of the Kolmogorov distribution $P_{K}\left(x\right)$ associated with the transition from A to *I*_1_ (see Figure S13). The dominant ensemble of paths passes via TS_1_ (see Figure 5B), with an interchange mechanism in which the distance between Ca^2+^ and CO_3_^2–^ increases, facilitating the arrival of a new water molecule. Instead, another less probable path goes via TS_2_ (see Figure 5B) and can be described as an associative substitution in which both carbonate oxygens remain coordinated, while the calcium-solvating waters temporarily adopt an eight-coordinated geometry.

It is interesting to note that this picture is strengthened by an analysis of the representation learned by the trained GNN. In fact, the attention mechanism identifies the water molecules that are most relevant to the Ca^2+^ coordination (see Section 2 for details). In Figure 5C, we plot together, as a function of the Ca–O distance, the pair correlation function and the attention weight distribution. Unsurprisingly, we see that the primary hydration shell at 2.3 Å is identified as highly important, but that a secondary peak at 2.9 Å is also relevant. This peak is related to the water molecules that take part in the TS_1_ ligand-exchange processes, discussed earlier. We also observe that the attention distribution starts to be different from zero and rather large as soon as the pair correlation is different from zero, reflecting the fact that the solvation waters at the smaller Ca–O distances are more effective at screening the cation and that such an effect is correctly encoded in the attention distribution.

## IV Discussion

The combination of our recent committor-based approach with the expressivity and generality of the GNN architectures offers a new, powerful tool for the semiautomatic study of rare events. This approach is descriptor-free, and the graph-based architecture provides new and powerful analysis tools to dissect in detail the reactive process under study. Such possibilities range from a simpler identification of relevant atoms, thanks to the one-to-one correspondence between reacting atoms and graph nodes, to the use of the model learned hidden representation as the basis for further analysis or the direct study of the information encoded into the attention layers.

Furthermore, we have also shown that the committor iterative optimization procedure^2,3^ can be improved and accelerated if one uses data from preliminary imperfect enhanced sampling simulations. In addition, we believe that our proposed suggestion to reduce the computational cost associated with the use of GNNs for enhanced sampling will help make its routine use more accessible, as it has happened in other fields, such as the construction of interatomic potentials.

## Supplementary Material



## Data Availability

The code for the training of the NN-based committor model alongside didactic tutorials is available through the open-source mlcolvar library,^41^ which is the preferred way to access the most updated code. Training and simulation data and inputs are available on https://github.com/alphatestK/GNN-Committor.

## Notes

Details of the truncated graph construction; additional computational details and materials for the examples in the main text; an additional example: Chignolin folding (PDF)
