# Supplementary material for: Committors without Descriptors
Source: J Chem Theory Comput. 2026 Feb 11;22(4):1613–20. doi: 10.1021/acs.jctc.5c01848 (PMC12937106; doi:10.1021/acs.jctc.5c01848)
Supplement: Supplementary file 1 [file ct5c01848_si_001.pdf]

Supporting Information for:
Committors without Descriptors

Peilin Kang,[1,*] Jintu Zhang,[1,2,*] Enrico Trizio,[1] TingJun Hou*,[2,3,†] and Michele Parrinello*[1,‡]

[1] *Atomistic Simulations, Italian Institute of Technology, 16156 Genova, Italy*
[2] *Innovation Institute for Artificial Intelligence in Medicine of Zhejiang University, College of Pharmaceutical Sciences, Zhejiang University, Hangzhou 310058 Zhejiang, China*
[3] *State Key Lab of CAD&CG, Zhejiang University, Hangzhou, Zhejiang 310058, China*

* These authors contributed equally: Peilin Kang and Jintu Zhang

† tingjunhou@zju.edu.cn
‡ michele.parrinello@iit.it

I. DETAILS ABOUT THE SCHNET-GNN BASED ON TRUNCATED GRAPH.

As we have introduced in the main text, to reduce the computational cost of the GNN used in our committor model, we designed the so-called truncated graph method, which predict the committor value only using atoms near the reaction center of the investigated process. Briefly speaking, during the construction of the graph, we first build an “atom cluster” composed of “system” atoms and “environment” atoms within a specified radius from the reacting atoms, then generate input graph data using only atoms within such a truncated atom cluster. In the training stage, this procedure is performed for each entry in the dataset, whereas during MD propagation, we dynamically update the indices of the environment atoms included in the cluster. As a result, the number of atoms within the graph can fluctuate during simulation. To avoid sudden changes in the committor value caused by environment atoms entering or leaving the graph, we build a buffer region, as often done with neighbor lists. In practice, we truncate the system using a radius slightly larger than the graph cutoff radius (R_c), extended by a buffer size (Δ_b). Therefore, when an entering or leaving event occurs, the corresponding environment atom is not linked to the reacting atoms by any direct edge.

However, even with such a procedure, entering or leaving atoms will nevertheless contribute to the committor value, since there are still edges between such atoms and other environment atoms inside the graph. Thus, we carried out the following two modifications to the SchNet-GNN. First, we added a cosine cutoff function f^c to the internode message calculation process, and the new message function reads as follows:

$$\mathbf{m}_{ij} = \boldsymbol{W}(\mathbf{h}_j) f_\theta^F(\mathrm{RBF}(d_{ij}))\, f^c(d_{ij}) \quad \text{with} \quad f^c(d) = \begin{cases} [1+\cos(\pi d/R_c)]/2, & d < R_c \\ 0. & d \geq R_c \end{cases} \tag{1}$$

Then, more importantly, we used only the features of the system nodes in the pooling function to predict the committor value:

$$z = f_\theta^R \left(\frac{1}{N_s} \sum_{i \in \mathcal{S}} \mathbf{h}_i \right), \tag{2}$$

where $\mathcal{S}$ stands for the set of system nodes and N_s is the number of system nodes. Through the message-passing mechanism, when the GNN model has more than one layer, the system node features still carry information from the environment atoms and therefore reflect the overall structure of the truncated atom cluster. However, due to the action of the cutoff function f^c, environment atoms that are far from reaction atoms will contribute less to the model output. Especially, messages from atoms inside the buffer region will be weakened twice before they are merged into the system node features, and thus influence even less the model output. That is, by employing both the decay function and the selective pooling operation, we implemented the truncated graph-based GNN models in a smooth manner.

To demonstrate the action of the above procedure, we plot the maximum sensitivity of water oxygen atoms in the $CaCO_3$ system as a function of their distances from the solute in Fig. S1. As can be seen, as the solute-solution distance approaches the graph cutoff distance, positions of the corresponding solution atom contribute less and less to the predicted committor value. For solution atoms inside the buffer region, their sensitivity indicates that they are in fact negligible to the committor model. As a result, the implemented GNN-based committor can be truly smooth when a solution atom enters or leaves the truncated graph, and thus is suitable for biased enhanced sampling simulations.

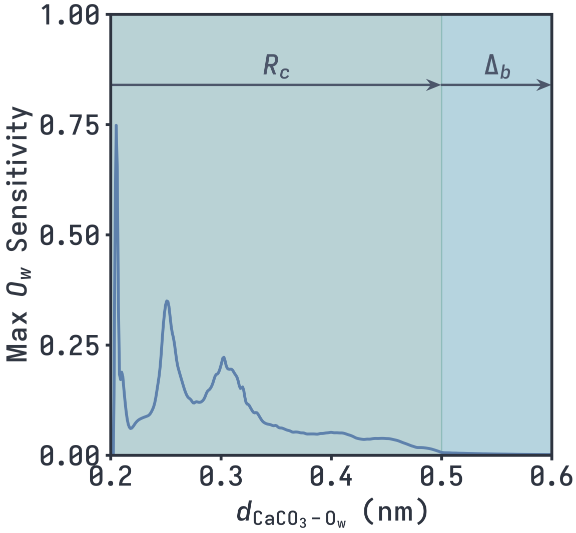

Figure S1. Maximum sensitivity of water oxygen atoms in the $CaCO_3$ system as a function of their shortest distances from the ion pair.

II. ALANINE DIPEPTIDE - ADDITIONAL INFORMATION

A. Computational details

Simulations details We utilized the AMBER99SB [1] force field to describe the alanine dipeptide molecule (Ace-Ala-Nme) in a vacuum. The lengths of all chemical bonds involving hydrogen atoms were constrained at their equilibrium values using the LINCS [2] algorithm. The electrostatic and Lennard-Jones (LJ) interactions were described in a non-cutoff manner. All MD propagations were carried out under the *NVT* ensemble using the GROMACS v2021.5 [3] package with the Langevin integrator [4], in combination with the PLUMED [5, 6] plugin. The integration time step and the friction coefficient were set to 2 fs and 20 ps^{-1}, correspondingly.

Committor model training details To model the committor function $q_\theta(\mathbf{x})$ at each iteration, we employed all 10 heavy atoms as inputs to the graph neural network. Since the system does not include solvent, no distinction between core and environment atoms was necessary here. The graph cutoff radius R_c is selected as 1.0 nm. The GNN architecture consisted of three layers, with 16 Gaussian basis functions, an output feature dimensionality of 20, and a two-layer feed-forward readout network. For message aggregation, we used the minimum-value operation.

For the optimization, we used the ADAM optimizer with an initial learning rate of $4 * 10^{-4}$ modulated by an exponential decay with multiplicative factor $\gamma = 0.99993$. The training was performed for 200 epochs in the first iteration and for ~500 epochs for the others. The α hyperparameter in the loss function was set to 10. The number of iterations, the corresponding dataset size, and the λ and the OPES `BARRIER` used in the biased simulations are summarized in Table S1 alongside the lowest value obtained for the functional K_m, which provides a quality and convergence measure, the simulation time t_s and the output sampling time t_o.

We typically terminate the iterative procedure when the change in Km between successive iterations is below 20%. To test the robustness of the GNN architecture, in this example, we used only unbiased data for the initial guess and did not employ the fast-converging OPES+$V_\mathcal{K}$ strategy; instead, OPES and $V_\mathcal{K}$ biased trajectories were run separately. The training is performed on an NVIDIA A100 GPU, and each iteration takes approximately one hour.

Simulation Speed Since all examples in this paper are performed using classical force fields, the relative computational overhead of GNN-based biasing is significant, as we report below. We also note that when the potential energy surface is evaluated using higher-level methods — such as DFT, QM/MM, or even Machine Learning potentials — the additional cost introduced by the GNN becomes much less significant. The biased simulation based on the GNN committor runs at a speed of 50 ns/day on an NVIDIA Quadro RTX 4000.

TABLE S1. Summary of the iterative procedure for Alanine.

Iteration	Dataset size	K_m [au]	OPES `BARRIER` [kJ/mol]	λ	t_s [ns]	t_o [ps]
0	20000	10180	-	-	2*10	1
1	50000	132	35	0.8	2*20	1
2	80000	10.3	35	0.8	2*20	1
3	110000	2.19	35	0.8	2*20	1
4	140000	2.09	35	0.8	2*20	1
5	170000	1.75	35	0.8	2*20	1

B. Additional figures

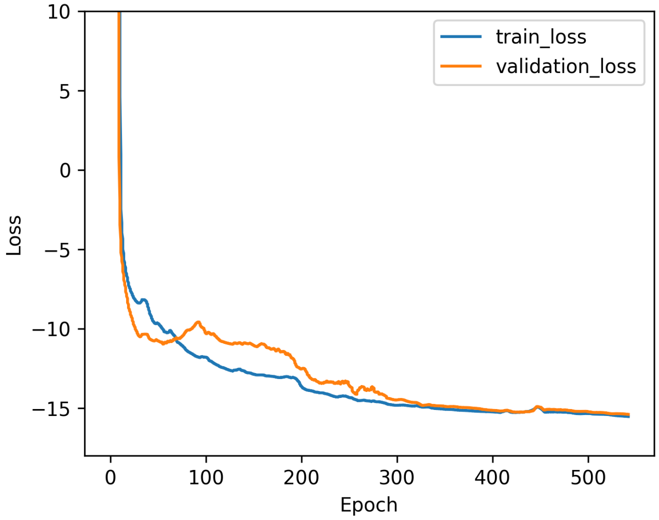

Figure S2. **Additional information of the committor of alanine dipeptide**: Training behavior in the final training iteration. The blue curve shows the training loss, while the validation loss is shown by the orange curve. The boundary loss contributions from basins A and B are both below 10^{-5}.

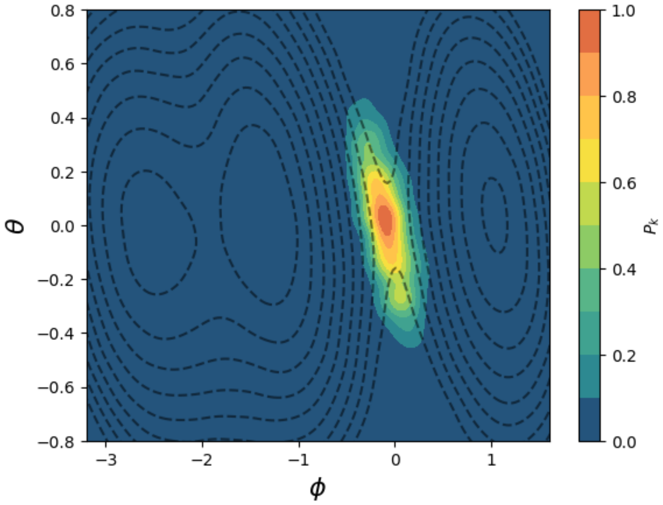

Figure S3. **Additional information of the committor of alanine dipeptide**: $p_{\mathcal{K}}$ distribution projected on φ and θ.

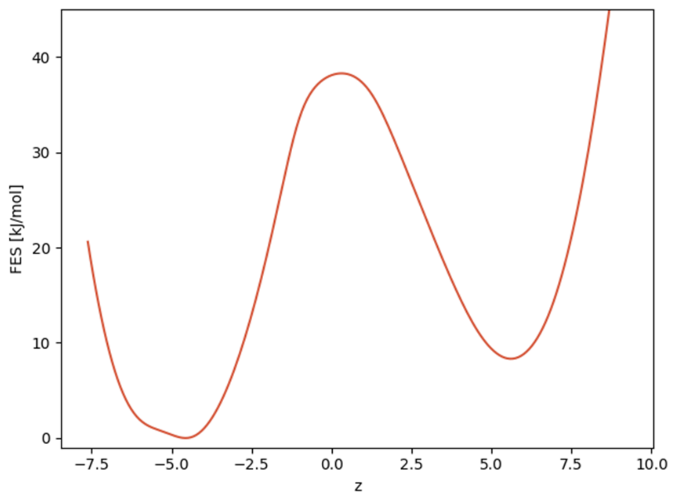

Figure S4. **Additional information of the committor of alanine dipeptide**: One dimensional Free energy surface projected on z.

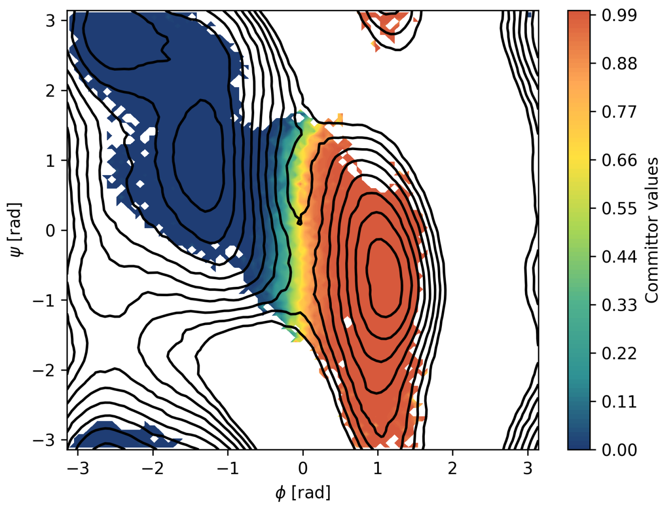

Figure S5. **Additional information of the committor of alanine dipeptide**: Two dimensional committor map projected on backbone dihedral angles φ and ψ. The black isolines of the free energy surface are superimposed as a reference. The results are consistent with our previous work using explicit descriptors[7, 8] as well as with those obtained using other methods reported in Ref. [9].

III. CALIXARENE - ADDITIONAL INFORMATION

A. Computational details

Simulations details We utilized the Amber General Force Field (GAFF) [10] with the RESP [11] charges and the TIP3P [12] water model to describe the Calixarene system. In our setup, a pair of OAMe host molecule and G_2 guest molecule was solvated at a density of about 1 g/cm^3 with 2100 water molecules in a cubic box of 4.027 × 4.027 × 4.027 nm^3. Nine extra sodium ions are included to counterbalance excess charges. The lengths of all chemical bonds involving hydrogen atoms were constrained at their equilibrium values using the LINCS [2] algorithm. Electrostatic interactions were described using the Particle Mesh Ewald (PME) [13] method with a real-space cutoff at 1.0 nm. The Lennard-Jones (LJ) interactions were calculated with a distance cutoff of 1.0 nm. All MD propagations were carried out under the *NVT* ensemble using the GROMACS v2021.5 [3] with the Langevin integrator [4] in combination with the PLUMED [5, 6] plugin. The integration time step and the friction coefficient were set at 2 fs and 1 ps^{-1}, correspondingly. At every simulation step, the coordinates are aligned so that the vertical axis of the box coincides with the binding axis h, and the simulation box is centered on the virtual atom V_1.

The funnel restraint In our simulations, we used a funnel restraint [14] equivalent to the one previously employed by Refs.15–17 on the same system. Here, we summarize the details of such a restraint, while more details can be found in the original works. The funnel limits the space available to the ligand in the unbound state U by confining it to a cylindrical volume above the binding site. As the ligand approaches the binding site, the funnel restraint becomes wider so that its presence does not affect the binding process itself. Having aligned with PLUMED the system to a reference configuration where the binding axis is found along the vertical axis, we define h as the projection on the binding axis of the center of the carbon atoms of each ligand and r as its radial component. When $h > 10$Å, the funnel surface is a cylinder with radius $R_{cyl} = 2$Å with its axis along the vertical direction. When $h < 10$Å, the funnel opens into an umbrella-like shape with a 45 degree angle whose surface is defined by $r = 12 - h$. The force that, for a displacement x, pushes the ligand away from the funnels surface is harmonic $-k_F x$ with $kF = 20$ kJ/mol Å^{-2}. A further harmonic restraint is applied on h to prevent the ligand from getting too far from the host, reaching the upper boundary of the simulation box. The corresponding force is $-k_U(h-18)$ for $h > 18$Å and $k_U = 40$ kJ/mol Å^{-2}.

During training, we set boundaries further to state U so that the labeled configurations used to impose the boundary conditions will not influence the committor training in the subsequent iterative simulations. We apply the funnel restraint described above and two additional harmonic restraints $-k_U(h-20)$ for $h > 20$Å and $-k_U(h-18)$ for $h < 18$Å, with $k_U = 20$ kJ/mol.

Because of the funnel presence, the free energy difference between the bound and the true unbound state that we extract from enhanced sampling simulations needs a correction that can be calculated as:

$$\Delta G = -\frac{1}{\beta}\log\left(C_0\pi R_{cyl}^2\int_B dh \exp\left[-\beta\left(W(h) - W_U\right)\right]\right) \tag{3}$$

where $\beta = 1/(k_B T)$, $C_0 = 1/1660$ Å^{-3} is the standard concentration, h is the coordinate along the funnels axis, $W(h)$ is the free energy along the funnel axis and W_U its reference value in state U. More precisely, we define W_U as the average free energy value in the interval 1.6 Å $< h <$ 1.8 Å. The integral is computed over the state B region that we define as 0.3 Å $< h <$ 0.8 Å.

Committor model training details To model the committor function $q_\theta(x)$ at each iteration, we used 4 atoms on the guest molecule and 12 atoms on the host skeleton as the reacting atoms of the system(see the labeled yellow atoms in Fig S6), and the neighboring oxygen atoms of water as environmental atoms. The cutoff radius were set to $R_c = 5$ Å and $R_b = 6$ Å. The GNN architecture consisted of two layers, with 12 Gaussian basis functions, an output feature dimensionality of 16, and a two-layer feed-forward readout network. For message aggregation, we used the attention mechanism described in the Method part.

For the optimization, we used the ADAM optimizer with an initial learning rate of $8 * 10^{-4}$ modulated by an exponential decay with multiplicative factor $\gamma = 0.9999$. The training was performed for 1000 epochs in the first iteration and for ~3000 epochs for the others. The training is performed on an NVIDIA A100 GPU, and each iteration takes approximately eight hours.

Simulation Speed The biased simulation based on the GNN committor runs at a speed of 30 ns/day on an NVIDIA Quadro RTX 4000.

TABLE S2. Summary of the iterative procedure for Calixarene.

Iteration	Dataset size	K_m [au]	OPES BARRIER [kJ/mol]	λ	t_s [ns]	t_o [ps]
0	100000	2250	50	-	100	1
1	120000	254.9	50	0.6	100	1
2	120000	23.5	50	0.6	100	1
3	120000	159	50	0.6	100	1
4	120000	5.99	50	0.6	100	1
5	120000	8.11	50	0.6	100	1

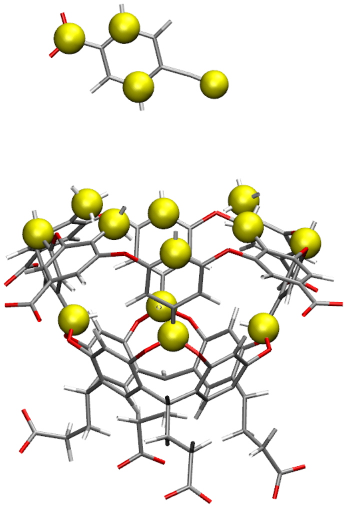

Figure S6. **Calixarene atoms set in the GNN architecture**: the yellow atoms represent the selected reacting atoms

B. Additional results

Training Behavior

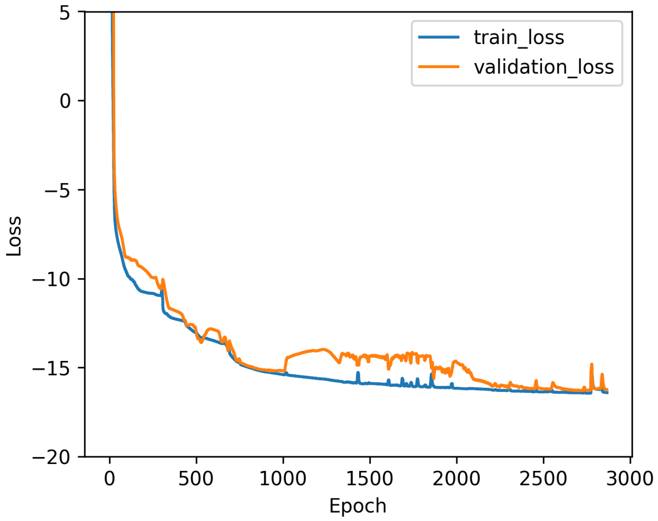

Figure S7. **Training behavior in the final training iteration.** The blue curve shows the training loss, while the validation loss is shown by the orange curve. The boundary loss contributions from basins A and B are both below 10^{-5}.

Free energy surface

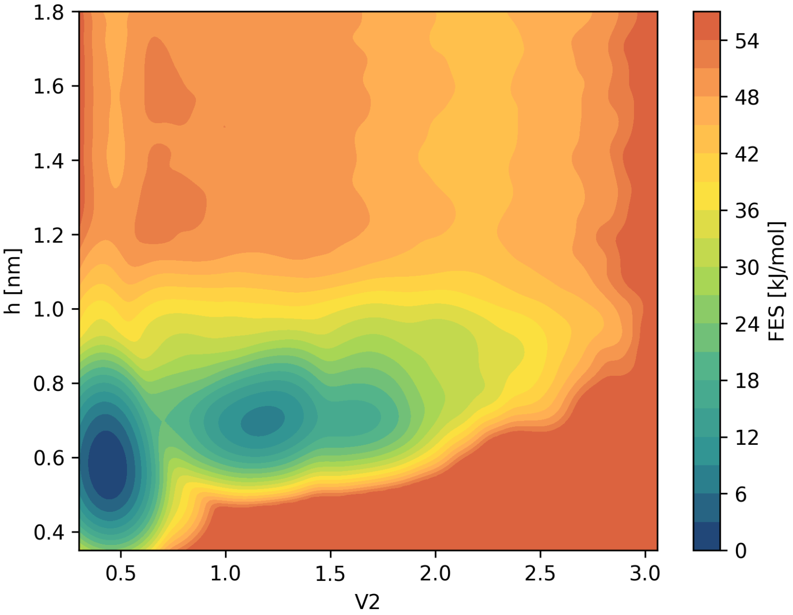

Figure S8. **2D free energy surface calixarene** Two-dimensional free energy surface for calixarene projected in the plane defined by the water coordination number of a virtual point inside the binding cavity (V_2) and the projection of the ligand molecule on the binding axis (h).

ΔF Convergency

The snapshots of two pathways

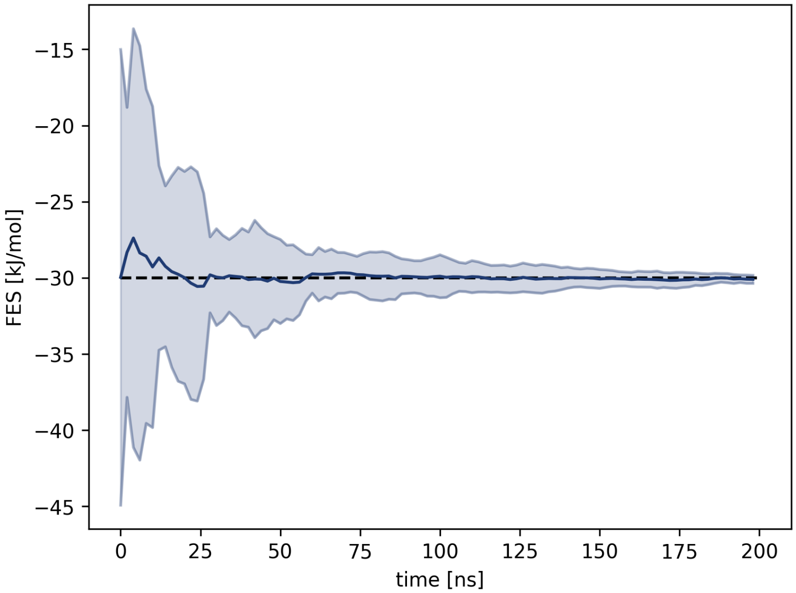

Figure S9. **Convergence with simulation time of the binding energy of the G_2 ligand to the OAMe octa-acid guest.** The average estimates from 3 independent simulations are reported as a blue solid line, whereas the uncertainty, computed as the standard deviation over the three replicas, is depicted as a shaded blue region. The reference values are provided as a black dashed line, and the reference estimate is obtained using the enhanced sampling setup of Ref. 15

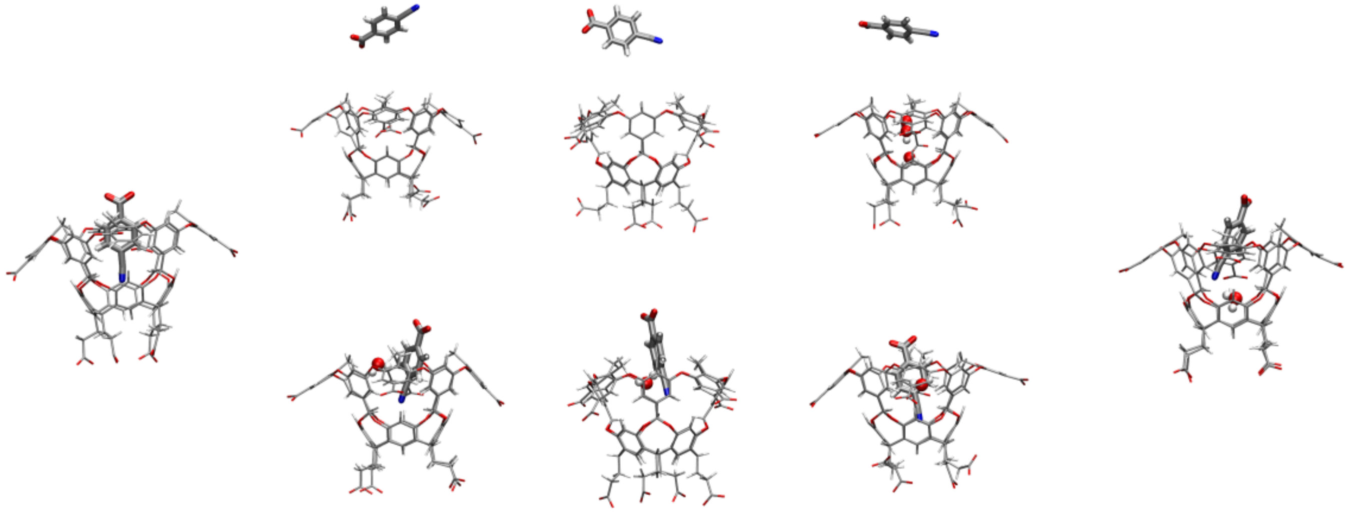

Figure S10. **Calixarene snapshots** Snapshots of representative configurations along the reaction pathway of B_d to B_w, grouped according to the z value. Water molecules inside the pocket are highlighted, while external waters are omitted for clarity. The upper pathway corresponds to a highly unlikely theoretical transition via the unbinding state, whereas the lower pathway represents the dominant route discussed in the main text.

IV. NaCl - ADDITIONAL INFORMATION

A. Computational details

Simulations details We utilized the force field parameters proposed by Joung and Cheatham [18] along with TIP3P [12] waters to describe the NaCl solution system. In our setup, a pair of NaCl ions was solvated at a density of about 1 g/cm^3 with 216 water molecules in a cubic box of 1.86 × 1.86 × 1.86 nm^3. The lengths of all H—O bonds were constrained at their equilibrium values using the RATTLE [19] algorithm. Electrostatic interactions were described using the Particle Mesh Ewald (PME) [13] method with a real-space cutoff at 0.9 nm. The Lennard-Jones (LJ) interactions were calculated with a distance cutoff of 0.9 nm, All MD propagations were carried out under the *NVT* ensemble using the OpenMM [20] package with the Geodesic BAOAB Langevin integrator [21] provided by OpenMMTools, [22] in combination with the PLUMED [5, 6] plugin. The integration time step and the friction coefficient were set at 2 fs and 1 ps^{-1}, correspondingly. The number of geodesic drift steps was set to 4.

Committor model training details To model the committor function $q_\theta(\mathbf{x})$ at each iteration, we used two ions Na$^+$ and Cl$^-$ as the reacting atoms of the system and the neighboring oxygen atoms of water are considered as environment atoms. The graph cutoff radius R_c is 0.6 nm and the buffer size Δ_b is 0.1 nm. The GNN architecture consisted of three layers, with 14 Gaussian basis functions, an output feature dimensionality of 24, and a two-layer feed-forward readout network. For message aggregation, we used the minimum-value operation. For the optimization, we used the ADAM optimizer with an initial learning rate of $4 * 10^{-4}$ modulated by an exponential decay with multiplicative factor $\gamma = 0.9999$. The training was performed for 1000 epochs in the first iteration and for ~2000 epochs for the others. The training is performed on an NVIDIA A100 GPU, and each iteration takes approximately four hours.

Simulation Speed The biased simulation based on the GNN committor runs at a speed of 36 ns/day on an NVIDIA Quadro RTX 4000.

TABLE S3. Summary of the iterative procedure for NaCl

Iteration	Dataset size	K_m [au]	OPES `BARRIER` [kJ/mol]	λ	t_s [ns]	t_o [ps]
0	38000	3.25	-	-	5	1
1	68000	2.72	15	0.2	16	0.5

TABLE S4. Summary of the iterative procedure for NaCl starting from only unbiased data

Iteration	Dataset size	K_m [au]	OPES `BARRIER` [kJ/mol]	λ	t_s [ns]	t_o [ps]
0	10000	23.3	-	-	5	1
1	40000	3.13	15	0.2	16	0.5
2	70000	2.89	15	0.2	16	0.5

B. Additional results

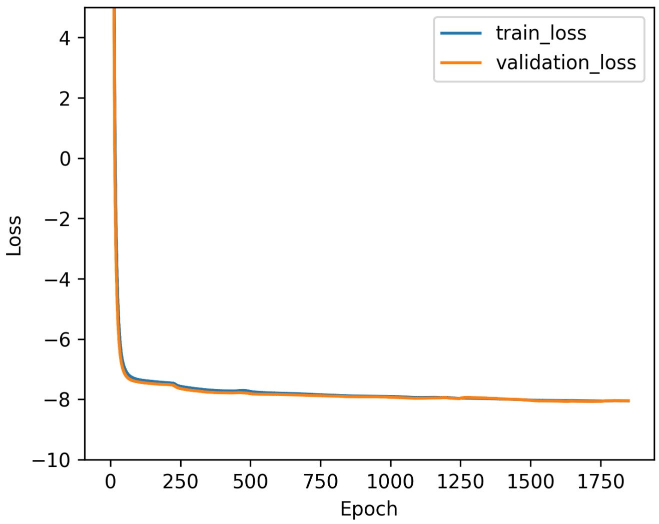

Figure S11. **Training behavior in the final training iteration.** The blue curve shows the training loss, while the validation loss is shown by the orange curve. The boundary loss contributions from basins A and B are both below 10^{-5}.

V. $CaCO_3$ - ADDITIONAL INFORMATION

A. Computational details

Simulations details We utilized the force field parameters for both ions and water proposed by Armstrong *et al.* [23] to describe the $CaCO_3$ solution system. In our setup, a pair of $CaCO_3$ ions was solvated at a density of about 1 g/cm^3 with 818 water molecules in a cubic box of 2.9011 × 2.9011 × 2.9011 nm^3. Electrostatic interactions were described using the Particle Mesh Ewald (PME) [13] method with a real-space cutoff at 0.9 nm. The Lennard-Jones (LJ) interactions were calculated with a distance cutoff of 0.9 nm. Specifically, the long-range dispersion correction to the LJ interactions was not applied, as required by the force field [23]. All MD propagations were carried out under the *NVT* ensemble using the OpenMM [20] package with the BAOAB Langevin integrator [24] provided by OpenMMTools, [22] in combination with the PLUMED [5, 6] plugin. The integration time step and the friction coefficient were set at 1 fs and 1 ps^{-1}, correspondingly.

Committor model training details

To model the committor function $q_\theta(x)$ at each iteration, we defined the ions Ca^{2+} and ${CO_3}^{2-}$ as the reacting atoms of the system, while the neighboring oxygen atoms of water were treated as environment atoms. The graph cutoff radius R_c is 0.6 nm and the buffer size Δ_b is 0.1 nm. In addition, to account for the large separation between ions upon full dissociation, we imposed fixed edges between the Ca^{2+} cation and all atoms belonging to the ${CO_3}^{2-}$ anion. The GNN architecture consisted of two layers, with 12 Bessel basis functions, an output feature dimensionality of 16, and a two-layer feed-forward readout network. For message aggregation, we used the attention mechanism described in the Method part.

For the optimization, we used the ADAM optimizer with an initial learning rate of 10^{-3} modulated by an exponential decay with multiplicative factor $\gamma = 0.9999$. The training was performed for 1000 epochs in the first iteration and for ~3000 epochs for the others. The training is performed on an NVIDIA A100 GPU, and each iteration takes approximately eight hours.

Simulation Speed The biased simulation based on the GNN committor runs at a speed of 21 ns/day on an NVIDIA Quadro RTX 4000.

TABLE S5. Summary of the iterative procedure for $CaCO_3$.

Iteration	Dataset size	K_m [au]	OPES `BARRIER` [kJ/mol]	λ	t_s [ns]	t_o [ps]
0	45000	4.99	25	-	30	1
1	34000	3.92	25	0.4	15	1
2	68000	2.90	25	0.4	30	1

B. Additional results

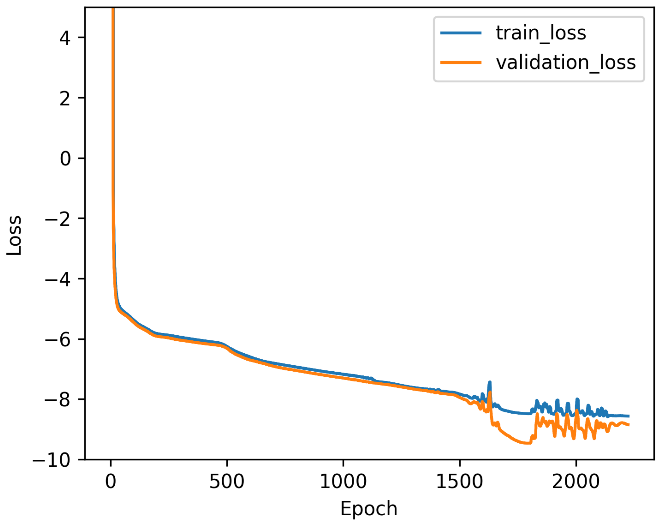

Figure S12. **Training behavior in the final training iteration.** The blue curve shows the training loss, while the validation loss is shown by the orange curve. The boundary loss contributions from basins A and B are both below 10^{-5}.

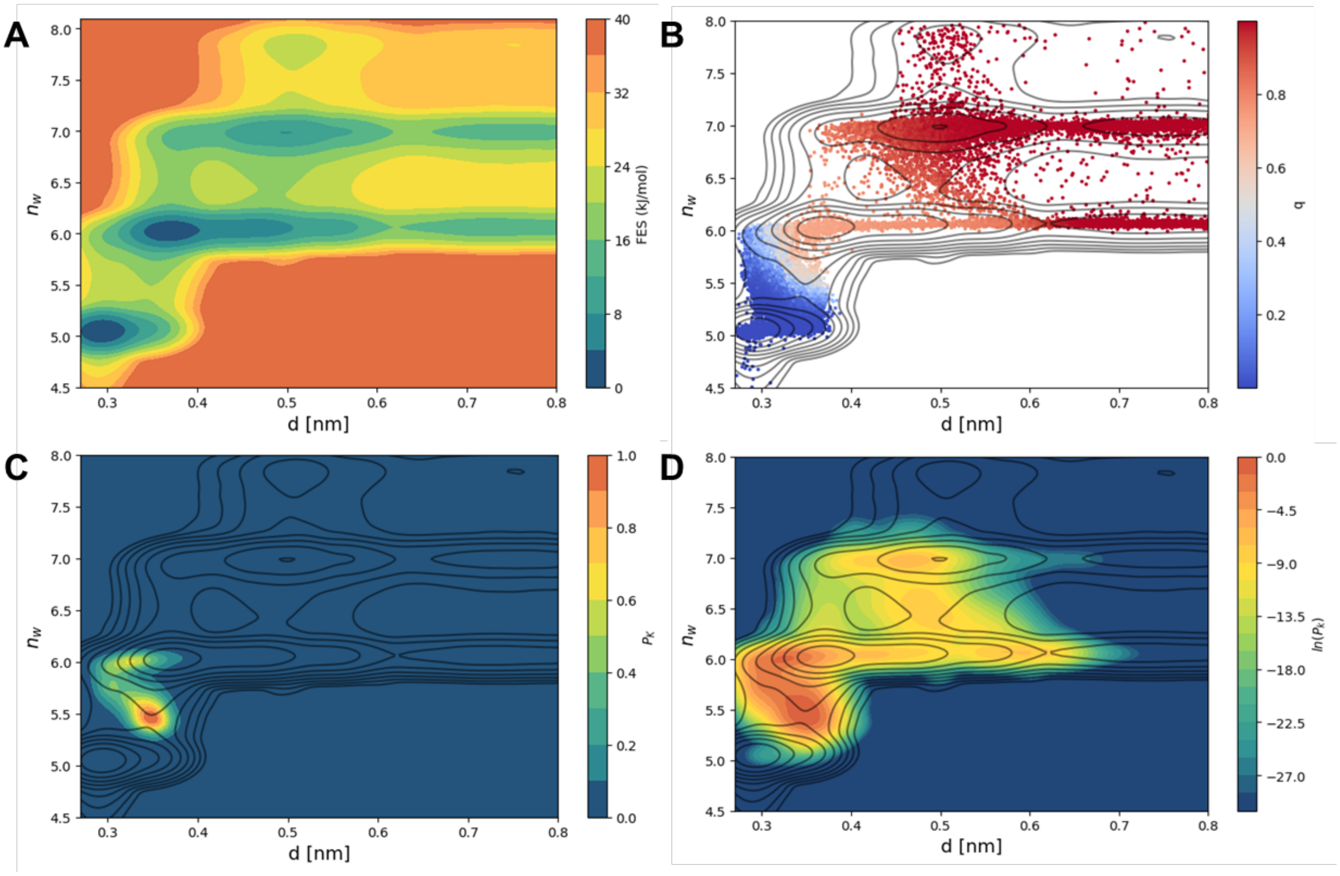

Figure S13. **Additional information of the committor of $\mathbf{CaCO_3}$ dissociation**:A) 2D FES projected on d and n_w B) Simulated dataset colored by committor value. C) $p_{\mathcal{K}}$ distribution projected on d and n_w. D) $\log(p_{\mathcal{K}})$ distribution projected on d and n_w. to show more details of the $p_{\mathcal{K}}$ distribution in the second barrier

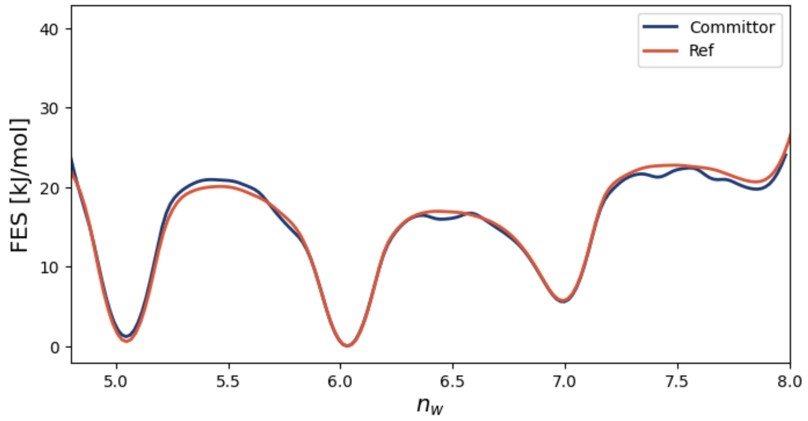

Figure S14. **1D FES of $CaCO_3$ dissociation process projected on** $\mathtt{n}_w$: The committor estimate (blue) is obtained from our OPES+$V_{\mathcal{K}}$ simulation biased on committor, and the reference estimate(red) is obtained from a long OPES simulation biased on $\mathtt{d}$ and $\mathtt{n}_w$

VI. FOLDING OF CHIGNOLIN

To assess the robustness and scalability of the method, we include an example of the folding of solvated chignolin, a small protein, into its stable hairpin structure. For this system, long unbiased MD simulations are available [25], and we therefore directly use these trajectories as the training dataset. However, even in these long trajectories, unfolding events remain relatively rare, making the training procedure less straightforward than one might anticipate. Nevertheless, the method still yields good results.

A. Computational details

Simulations details For the study of folding and unfolding of chignolin (CLN025 peptide sequence Tyr-Tyr-Asp-Pro-Glu-Thr-Gly-Thr-Trp-Tyr) in explicit solvent, we performed our simulations using GROMACS v2021.5 [3] patched with PLUMED, [5, 6] the CHARMM22* [26] force field, and the solvent has been modeled by the CHARMM TIP3P [27] force field, sharing the same setup used for long unbiased simulations on this system [25] to have a direct comparison with those results. For the same reason, we kept the simulation condition consistent with that work. All simulations were performed with an integration time step of 2 fs and sampling NVT ensemble at 340K. Asp, Glu residues, as well as the N- and C-terminal amino acids are simulated in their charged states. The simulation box contains 1,907 water molecules, together with two sodium ions that neutralize the system. The linear constraint solver algorithm is applied to every bond involving H atoms, and electrostatic interactions are computed via the particle mesh Ewald scheme, with a cutoff of 1 nm for all nonbonded interactions.

Committor model training details To model the committor function $q_\theta(\mathbf{x})$, we defined C_α atoms as the reacting atoms of the system, while the neighboring heavy atoms of chignolin were treated as environment atoms. The graph cutoff radius R_c is 0.6 nm and the buffer size Δ_b is 0.1 nm. In addition, to account for the long-distance skeletal movements, we imposed fixed edges between the C_α atoms. The GNN architecture consisted of two layers, with 16 Bessel basis functions, an output feature dimensionality of 20, and a two-layer feed-forward readout network. For message aggregation, we used the attention mechanism described in the Method part.

For the optimization, we used the ADAM optimizer with an initial learning rate of 10^{-3} modulated by an exponential decay with multiplicative factor $\gamma = 0.9999$. The training was performed ~2000 epochs. The training is performed on an NVIDIA A100 GPU, and each iteration takes approximately eight hours.

Simulation Speed The biased simulation based on the GNN committor runs at a speed of 26 ns/day on an NVIDIA Quadro RTX 4000.

B. Additional results

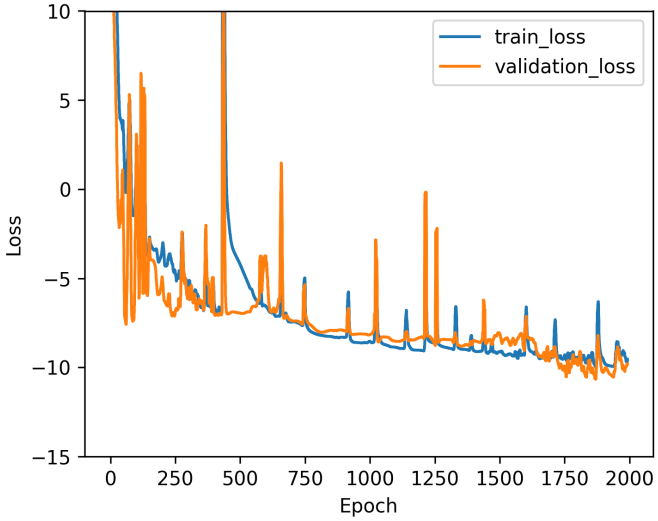

Figure S15. **Training behavior in the final training iteration.** The blue curve shows the training loss, while the validation loss is shown by the orange curve. The boundary loss contributions from basins A and B are both below 10^{-5}. The training process exhibits reduced stability compared with other examples because we use long unbiased trajectories as the training set, rather than a dataset enriched with transition-state configurations generated by our iterative procedure.

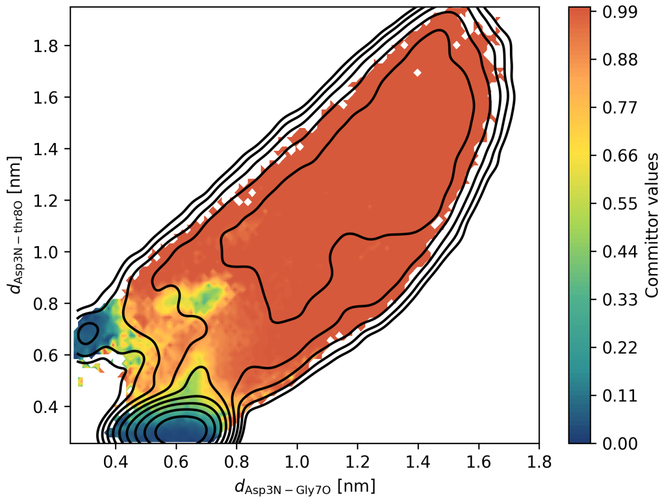

Figure S16. Two dimensional committor map projected on the distances between $\mathrm{Asp3N}-\mathrm{Tyr7O}$ and $\mathrm{Asp3N}-\mathrm{Thr8O}$. The black isolines of the free energy surface are superimposed as a reference. The results are consistent with the result obtained using other methods reported in Ref. [28].

REFERENCES

---

[1] R. Salomon-Ferrer, D. A. Case, and R. C. Walker, An overview of the amber biomolecular simulation package, WIREs Computational Molecular Science **3**, 198 (2013), https://wires.onlinelibrary.wiley.com/doi/pdf/10.1002/wcms.1121.

[2] B. Hess, H. Bekker, H. J. C. Berendsen, and J. G. E. M. Fraaije, Lincs: A linear constraint solver for molecular simulations, J. Comput. Chem. **18**, 1463 (1997).

[3] M. J. Abraham, T. Murtola, R. Schulz, S. Páll, J. C. Smith, B. Hess, and E. Lindahl, Gromacs: High performance molecular simulations through multi-level parallelism from laptops to supercomputers, SoftwareX **1**, 19 (2015).

[4] S. Kieninger and B. G. Keller, Gromacs stochastic dynamics and baoab are equivalent configurational sampling algorithms, J. Chem. Theory Comput. **18**, 5792 (2022).

[5] G. A. Tribello, M. Bonomi, D. Branduardi, C. Camilloni, and G. Bussi, PLUMED 2: New feathers for an old bird, Computer physics communications **185**, 604 (2014).

[6] The PLUMED consortium., Promoting transparency and reproducibility in enhanced molecular simulations, Nat. Methods **16**, 670 (2019).

[7] P. Kang, E. Trizio, and M. Parrinello, Computing the committor with the committor to study the transition state ensemble, Nature Computational Science , 1 (2024).

[8] E. Trizio, P. Kang, and M. Parrinello, Everything everywhere all at once: a probability-based enhanced sampling approach to rare events, Nature Computational Science , 1 (2025).

[9] H. Chen, B. Roux, and C. Chipot, Discovering reaction pathways, slow variables, and committor probabilities with machine learning, Journal of Chemical Theory and Computation (2023).

[10] J. Wang, R. M. Wolf, J. W. Caldwell, P. A. Kollman, and D. A. Case, Development and testing of a general amber force field, Journal of computational chemistry **25**, 1157 (2004).

[11] C. I. Bayly, P. Cieplak, W. Cornell, and P. A. Kollman, A well-behaved electrostatic potential based method using charge restraints for deriving atomic charges: the resp model, The Journal of Physical Chemistry **97**, 10269 (1993).

[12] W. L. Jorgensen, J. Chandrasekhar, J. D. Madura, R. W. Impey, and M. L. Klein, Comparison of simple potential functions for simulating liquid water, J. Chem. Phys. **79**, 926 (1983).

[13] U. Essmann, L. Perera, M. L. Berkowitz, T. Darden, H. Lee, and L. G. Pedersen, A smooth particle mesh ewald method, J. Chem. Phys. **103**, 8577 (1995).

[14] V. Limongelli, M. Bonomi, and M. Parrinello, Funnel metadynamics as accurate binding free-energy method, Proceedings of the National Academy of Sciences **110**, 6358 (2013).

[15] V. Rizzi, L. Bonati, N. Ansari, and M. Parrinello, The role of water in host-guest interaction, Nature Communications **12**, 93 (2021).

[16] S. Bhakat and P. Söderhjelm, Resolving the problem of trapped water in binding cavities: prediction of host–guest binding free energies in the sampl5 challenge by funnel metadynamics, Journal of computer-aided molecular design **31**, 119 (2017).

[17] S. Pérez-Conesa, P. M. Piaggi, and M. Parrinello, A local fingerprint for hydrophobicity and hydrophilicity: From methane to peptides, The Journal of chemical physics **150** (2019).

[18] I. S. Joung and T. E. I. Cheatham, Determination of alkali and halide monovalent ion parameters for use in explicitly solvated biomolecular simulations, J. Phys. Chem. B **112**, 9020 (2008).

[19] H. C. Andersen, Rattle: A "velocity" version of the shake algorithm for molecular dynamics calculations, J. Comput. Phys. **52**, 24 (1983).

[20] P. Eastman, J. Swails, J. D. Chodera, R. T. McGibbon, Y. Zhao, K. A. Beauchamp, L.-P. Wang, A. C. Simmonett, M. P. Harrigan, C. D. Stern, R. P. Wiewiora, B. R. Brooks, and V. S. Pande, Openmm 7: Rapid development of high performance algorithms for molecular dynamics, PLoS Comput. Biol. **13**, e1005659 (2017).

[21] B. Leimkuhler and C. Matthews, Efficient molecular dynamics using geodesic integration and solventsolute splitting, Proc. Math. Phys. Eng. Sci. **472**, 20160138 (2016).

[22] J. Chodera, A. Rizzi, L. Naden, K. Beauchamp, P. Grinaway, J. Fass, I. Pulido, A. Wade, M. Henry, G. A. Ross, A. Krämer, H. B. Macdonald, jaimergp, B. Rustenburg, D. W. Swenson, I. Zhang, A. Simmonett, M. J. Williamson, hb0402, J. Fennick, S. Roet, SimonBoothroyd, A. Silveira, and D. Rufa, choderalab/openmmtools: 0.23.1 (2023), accessed on 2023-09-21.

[23] B. Armstrong, A. Silvestri, R. Demichelis, P. Raiteri, and J. D. Gale, Solubility-consistent force field simulations for aqueous metal carbonate systems using graphical processing units, Philos. Trans. R. Soc. A: Math. Phys. Eng. Sci. **381**, 20220250 (2023).

[24] B. Leimkuhler and C. Matthews, Rational construction of stochastic numerical methods for molecular sampling, Appl. Math. Res. eXpress **2013**, 34 (2012).

[25] K. Lindorff-Larsen, S. Piana, R. O. Dror, and D. E. Shaw, How fast-folding proteins fold, Science **334**, 517 (2011).

[26] S. Piana, K. Lindorff-Larsen, and D. E. Shaw, How robust are protein folding simulations with respect to force field parameterization?, Biophysical Journal **100**, L47 (2011).

[27] A. D. J. MacKerell, D. Bashford, M. Bellott, R. L. J. Dunbrack, J. D. Evanseck, M. J. Field, S. Fischer, J. Gao, H. Guo, S. Ha, D. Joseph-McCarthy, L. Kuchnir, K. Kuczera, F. T. K. Lau, C. Mattos, S. Michnick, T. Ngo, D. T. Nguyen, B. Prodhom, W. E. Reiher, B. Roux, M. Schlenkrich, J. C. Smith, R. Stote, J. Straub, M. Watanabe, J. Wiórkiewicz-

Kuczera, D. Yin, and M. Karplus, All-atom empirical potential for molecular modeling and dynamics studies of proteins, The Journal of Physical Chemistry B **102**, 3586 (1998), pMID: 24889800, https://doi.org/10.1021/jp973084f.

[28] G. Lazzeri, H. Jung, P. G. Bolhuis, and R. Covino, Molecular free energies, rates, and mechanisms from data-efficient path sampling simulations, Journal of Chemical Theory and Computation **19**, 9060 (2023).
